# Adaptation of a community-based type-2 diabetes mellitus remission intervention during COVID-19: empowering persons living with diabetes to take control

**DOI:** 10.1186/s43058-022-00255-9

**Published:** 2022-01-24

**Authors:** Kim R. Quimby, Madhuvanti M. Murphy, Heather Harewood, Christina Howitt, Ian Hambleton, Selvi M. Jeyaseelan, Natalie Greaves, Natasha Sobers

**Affiliations:** 1 The George Alleyne Chronic Disease Research Centre, Caribbean Institute for Health Research, The University of the West Indies, Jemmott’s Lane, St. Michael, Barbados; 2 The Faculty of Medical Sciences, The University of the West Indies, St. Michael, Barbados; 3 For Research Inc., St. Michael, Barbados

**Keywords:** Community intervention, Online intervention, Adaptation, Modification, Fidelity, Self-monitor, Low-calorie diet, T2DM remission, Weight loss, COVID-19

## Abstract

**Background:**

The Barbados Diabetes Remission Study-2 reported that a low-calorie diet for weight loss and diabetes remission implemented within the community and supported by trained community health advocates was both an acceptable implementation strategy and a clinically effective intervention. This study aimed to examine the adaptability of the face-to-face protocol into an online modality.

**Methods:**

The Iterative Decision-making for Evaluation of Adaptations (IDEA) framework guides researchers in examining the necessity of the adaptation and the preservation of core intervention elements during the adaptation process. Adaptation outcomes were documented using the Framework for Reporting Adaptations and Modifications to Evidence-Based Implementation Strategies (FRAME-IS). Implementation outcome was determined by fidelity to core elements. Intervention effectiveness was determined from the analysis of clinical data.

**Results:**

We decided that an adaptation was needed as COVID-19 control measures prohibited in-person interactions. The core elements—i.e. 12-week intervention duration, daily 840-kcal allowance, and weekly monitoring of weight and blood glucose—could be preserved during the adaptation process. Adaptations were made to the following: (1) the context in which data were collected—participants self-measured at home instead of following the original implementation strategy which involved being measured by community health advocates (CHA) at a community site; (2) the context in which data were entered—participants posted their measurements to a mobile application site which was accessible by CHAs. As with the original protocol, CHAs then entered the measurements into an online database; (3) the formulation of the low-calorie diet—participants substituted the liquid formulation for a solid meal plan of equivalent caloric content. There was non-inferiority in fidelity to attendance with the online format (97.5% visit rate), as compared to the face-to-face modality (95% visit rate). One participant deviated from the calorie allowances citing difficulty in estimating non-exact portion sizes and financial difficulty in procuring meals. Weight change ranged from − 14.3 to 0.4 kg over the 12-week period, and all group members achieved induction of diabetes remission as determined by a FBG of < 7mmol/l and an A1C of < 6.5%.

**Conclusion:**

The results suggest that this adapted online protocol—which includes changes to both the implementation strategy and the evidence-based practice—is clinically effective whilst maintaining fidelity to key elements. Utilization of the IDEA and FRAME-IS adaptation frameworks add scientific rigour to the research.

**Trial registration:**

ClinicalTrials.govNCT03536377. Registered on 24 May 2018

Contributions to the literature

- The parent study showed that a community-based low-calorie dietary intervention is acceptable and clinically effective at inducing weight loss and diabetes remission.
- Restrictions, because of the COVID-19 pandemic, prohibited this face-to-face format. In response, we implemented a modified protocol which was fully online and included participants self-monitoring at home and posting the results to an online platform, which also doubled as a social support network.
- Fidelity to the core elements of the intervention was maintained during the adaptation.
- The adapted protocol was acceptable and clinically effective at inducing weight loss and diabetes remission.

## Background

Care regimens that place the primary responsibility of disease monitoring on the healthcare system can be problematic as factors such as access, cost, and prolonged waiting room times act as deterrents to utilization [1, 2]. This is particularly true in low-resource settings where universal health coverage has not been fully realized. The impact of these hindrances to access was exacerbated during the COVID-19 pandemic as an estimated 49% of diabetes services were interrupted as clinical staff from noncommunicable diseases (NCD) clinics were reassigned to COVID-19 support roles [3]. Whilst some countries have instituted additional diabetes community clinics, this may not be possible in under-resourced areas, thereby compounding disparities in access [4]. As persons with diabetes are at risk for poorer clinical outcomes if infected with COVID-19, such patients may choose to avoid activities outside of the home including doctors’ visits, thereby challenging the concept of acceptability of the ambulatory care model in these times [5, 6]. Community-based interventions that leverage the use of virtual modalities may help to close the gap in access to care in settings where the Internet is accessible [7].

In this article, we discuss the adaptations made to the parent study described in the article entitled *The impact of a community-based low-calorie intervention on the induction of type-2 diabetes and pre-diabetes remission: a feasibility study utilizing a type-2 hybrid design* [8]. The implementation strategy was based on a community health advocate (CHA) training model, utilizing three faith-based organizations (FBOs) as community intervention sites. During the implementation phase, volunteer congregants participated in a 10-week course where they were taught to measure weight, blood glucose, and blood pressure (BP); this was followed by a mandatory, summative practical examination which determined their eligibility to perform the CHA role during the intervention phase. The intervention was a 12-week low-calorie diet of mainly the commercial liquid formulation Glucerna®. The daily caloric allowance was 840 kcal. Participants were persons who were diagnosed with T2DM for ≤ 6years or pre-diabetes and were overweight (BMI ≥ 27). During the 12-week low-calorie intervention phase, CHAs met with participants at the FBO on a weekly basis to measure the participants’ weight, fasting blood glucose (FBG), and BP. HbA1C was performed at weeks 1 and 12 only. During these weekly consultations, CHAs also had one-on-one discussions with participants regarding dietary (non)compliance over the past week. Participants were counselled as necessary. Communication between participants and CHAs continued during the week via group WhatsApp chats, which were divided by FBO site. Conversations were varied including daily devotions centred around the Christian faith, confessions of difficulty in conforming rigidly to the meal plan, encouragement and instructions from CHAs and fellow participants on how to avoid pitfalls, and general check-ins on each other’s well-being. We reported that this implementation strategy was acceptable to CHAs and participants and that the intervention under these circumstances was clinically effective at inducing weight loss and T2DM and pre-diabetes remission in a subset of the first cohort of 31 participants.

During the recruitment of the second cohort, the enrollment process was suspended due to the threat of COVID-19; however, the five participants that were already enrolled began the low-calorie diet. They were all previously diagnosed with T2DM and were all at a single FBO site. To continue the study during the anticipated national lockdown, these five participants were instructed on how to self-monitor for weight, FBG, and BP and provided with scales, glucometers, and BP kits as necessary. During week 3 of the 12-week intervention, the government instituted a “work from home” curfew for all non-essential services. Under these directives, all face-to-face contact on the study was aborted.

Here, we describe how the research protocol was adapted to overcome the barriers associated with the COVID-19 directives. The decision to adapt was guided by the Iterative Decision-making for Evaluation of Adaptations (IDEA) tool which included noting the need for the adaptation, determining if fidelity to key intervention components will be preserved and evaluating the intervention outcomes in comparison with the original protocol [9]. The adaptation outcomes were also documented, using the Framework for Reporting Adaptations and Modifications to Evidence-based Implementation Strategies (FRAME-IS); this records the reasons for and the nature of the adaptation process [10].

## Methods

Within the implementation science literature, the terms “adaptation” and “modification” have been used interchangeably. Some articles, however, have defined modifications as any change to the intervention whereas adaptations are restricted to those changes which were planned or proactive in nature [10]. Here, we used the terms as outlined by Moore et al.—“proactive adaptation” for those changes that were planned prior to the intervention phase and “reactive adaptation” for the changes that were made in response to unforeseen events [11].

### The decision to adapt

The decision to adapt was guided by six questions posed within the IDEA framework: (A) Is an adaptation needed? (B) Are the core elements of the intervention known? (C) Can the barrier be addressed whilst preserving the core elements? (D) Does the timeframe allow a pilot? (E) Are the outcomes non-inferior or improved? and (F) Is the “voltage drop” acceptable to stakeholders?

### Adaptation outcomes

The outcomes of the adaptations made during this study were characterized using the FRAME-IS, which comprises 4 core modules and 3 optional modules. The framework considers the following: module 1—the description of the intervention, the implementation strategy, and the modification; module 2—what was modified; module 3—(a) the nature of the modification and (b) if the modification maintained fidelity to the core elements; module 4—(a) the goal of the modification and (b) the level of the modification: module 5—(a) when the modification occurred and (b) if the adaptations were planned; module 6—who participated in the decision to modify; and module 7—how widespread the modification was [10].

### Implementation outcomes

Fidelity of CHAs to the 12-week intervention duration was determined from the attendance register and online activity. Fidelity to the weekly monitoring of participants for change in weight and fasting blood glucose was assessed from the weekly WhatsApp data entry of clinical measurements by participants and entry of this data into REDCap by the CHAs.

### Intervention outcomes

Participant compliance with the 12-week duration was determined by the attendance register and fidelity to the daily 840 kcal allowance was gauged from the weekly dietary notification and online conversations. Change in weight, fasting blood glucose, HbA1C, and the induction of T2DM remission were analysed from the participant data entries.

## Results

### The decision to adapt

In response to the questions posed by the IDEA:

(A) Is an adaptation needed? It was determined that an adaptation was necessary as the existing COVID-19-related public health policy prohibited the continuation of the intervention in the original face-to-face format.
(B) Are the core elements of the intervention known? The core elements—i.e. elements that are necessary to maintain the integrity of the intervention—were identified as (1) the 12-week duration of the study, (2) the weekly monitoring, and (3) the 840-kcal daily calorie consumption. This determination was based on the intervention effectiveness of previous studies which suggested that this paradigm of a very low-calorie diet for in excess of 8-week duration was sufficient to cause the quantity of weight loss needed to induce diabetes remission [12, 13]. Maintaining this paradigm also allows for the comparison of clinical outcomes with the previous cohort that underwent the intervention prior to this adaptation [8].
(C) Can the barrier be addressed whilst preserving the core elements? Study investigators decided that the adaptation, which would be contextual in nature (i.e. a change in the mode of intervention delivery whilst preserving the content of the intervention), can be implemented without compromising fidelity to the core intervention elements.
(D) Does the time frame allow a pilot? As the participants had already started the intervention in its original format, and the need to switch to the adapted version was immediate, study investigators determined that there was no time to pilot the adapted protocol.
(E) Are the outcomes non-inferior or improved?
(F) Is “voltage drop” acceptable to stakeholders?

As there was no pilot data to analyse, we could not determine the outcomes. Using this pragmatic approach, the decision was made to proceed with the adaptation and evaluate the outcomes.

### Adaptation outcomes

Adaptation outcomes were documented using the FRAME-IS. Utilizing the framework for Reporting Adaptations and Modifications to Evidence-Based Interventions (FRAME) yielded a similar description and hence is not duplicated in these results.

Module 1: The description of the intervention, the implementation strategy, and the modification—The intervention was a low-calorie diet aimed at inducing weight loss and diabetes remission in participants with overweight and T2DM. The implementation strategy was to train CHAs to support the intervention by performing participant weight and FBG measurements at the local FBO on a weekly basis. Adaptations were made to the context in which the participant measurements were performed to maintain the fidelity to the intervention during the COVID-19 pandemic.

Module 2: What was modified—All changes were intended to be made to the context in which the intervention was delivered, whilst preserving the content of the intervention (Table 1).

**Table 1:** Adaptation outcomes using the framework for reporting adaptations and modifications to evidence-based implementation strategies (FRAME-IS)

Goal of the modification	Who participated in the decision to modify?	What is modified? (nature of content modification)	Fidelity to core elements
To facilitate weekly monitoring of clinical measurements in the context of prohibited research-related face-to-face interactions	Principal investigator CHA Participants	Content: unchanged; clinical measurements were continued on a weekly basis. Context: changed. Original: clinical measurements conducted by the CHA at the FBO Adaptation: clinical measures performed by the participants at home.	Fidelity: consistent; during the face-to-face format, the visit rate was 95%. This is comparable with the previous cohort where the visit rate was 94.8%. During the adapted online format, the visit rate was 97.8%.
To facilitate the documentation of clinical measurements to allow the principal investigator to monitor the status	Principal investigator CHA Participants	Content: unchanged; clinical data was reviewed on a weekly basis. Context: changed. Original: data was entered by CHAs into the online REDCap database. Adaptation: data was posted by participants in the WhatsApp mobile application and then uploaded by the CHA to the REDCap database.	Fidelity: consistent; the ability of the PI to monitor hinged on the data entered (see row above).
To provide participants with an alternative to the liquid low-calorie diet formulation	Principal investigator CHA Participants Dietician	Content: unchanged; the daily calorie allowance remained at 840kcal/day. Context: changed Original: diet comprised mainly of a liquid formulation. Adaptation: diet comprised a mixture of solid and liquid formulation.	Fidelity: partially consistent. One participant experienced some difficulty both sourcing and preparing the solid low-calorie diet.

The first adaptation was made to the implementation strategy and involved changes to the context in which the clinical measurements were taken. In the original protocol, participants were measured on a weekly basis by trained CHA at the FBO community site. However, in the adapted version, participants were taught how to self-monitor and issued with the glucometers, scales, and BP machines as necessary; during week 4 of the intervention, participants started to self-monitor at home in accordance with the weekly protocol.

The second adaptation was also made to the implementation strategy and involved the way the data were collected and entered. Originally, clinical measurements were entered by CHAs into the online REDCap database on the day that participants came to be measured; the data were immediately accessible by the study PI. In the adapted protocol, participants self-monitoring at home posted their readings in the WhatsApp mobile application. As the PI was a member of the online group, the data were still immediately accessible. The designated CHA in the chat then entered the data into REDCap. Hence, the group chat adopted a dual function as it became a medium to report on progress in addition to being used to provide social support.

The third adaptation was to the clinical practice and involved a change to a low-calorie diet formulation. During week 8 of the intervention, the supply chain for the Glucerna® was interrupted. Online Zoom meetings with the study dietician were arranged within the week, and under her guidance, participants learned how to create nutritionally balanced solid meals of the same caloric content (840 kcal/day), using ingredients that were either locally produced or readily available.

Module 3 (a): The nature of the modification—The adaptations were substitutions, as the clinical measurements and reporting of these measurements were changed from being CHA-led to being participant-led. In addition, the liquid formulation was substituted with solid meals of similar caloric content. Module 3 (b): maintenance of fidelity to the core elements—the core elements of the intervention were preserved.

Module 4 (a): The goal of the modification—The goal of the adaptation was to allow participants to maintain fidelity to the intervention despite the national COVID-19 directives that disrupted the face-to-face protocol. Module 4 (b): the level of the modification—the level of the modification was at both the “teacher” and “recipient” level as the responsibility for monitoring and reporting was transferred from the CHA to the participant.

Module 5 (a): When the modification occurred—The decision to adapt was made during the intervention phase. The adaptation process was designed during week 1 of the intervention in response to the threat of COVID-19 infection to the island and enacted during week 4 in response to the national COVID-19 directives. Module 5(b): if the adaptations were planned—using the definition proposed by Moore et al., the change was classified as an unplanned or reactive adaptation [11]. If the FRAME-IS definition is used, the change would be classified as a planned/reactive modification or reactive adaptation [10].

Module 6: Who participated in the decision to modify—The decision to adapt was a participatory effort including the study team, CHAs, and the participants; however, the final decision was made by the study principal investigator (PI).

Module 7: How widespread was the modification—The adaptation covered all CHAs and participants who were currently enrolled in the programme.

### Implementation outcomes

#### CHA fidelity to the 12-week duration

Review of the attendance register and the online data showed that CHAs were in attendance on a weekly basis for the 12-week duration.

#### Fidelity to the weekly monitoring

During the 12-week intervention period, there were approximately 3000 total WhatsApp entries for these five participants, some 800 more than the previous group of seven participants who were managed at the same FBO site as a sub-group of the 31 participants who were governed by the original protocol. Posts were made almost daily with conversations initiated by both CHAs and participants. Most participants submitted their readings within the 24-h period; participants who could not submit on the scheduled day (e.g. because of work), took the initiative to reschedule to another day within the same week.

In the previous cohort (*n* = 31) which included participants from 3 FBO sites, there were 403 (31 participants × 13 weeks) potential visits, and 21 visits were not made, giving a visit rate of 94.8%. In this cohort (*n* = 5), during the 4 weeks prior to adaptation, there were 20 (5 × 4) potential visits, and 1 visit was not made, for a visit rate of 95%. After adaptation, this cohort then had 9 online entries to complete (to make up the anticipated 13 visits/entries). From a possible 45 entries (5 × 9), 44 complete entries were recorded, for a completion rate of 97.8%.

### Intervention outcomes

#### Participant fidelity to the 12-week duration

Review of the attendance register showed that all five participants remained in the study for the 12-week duration.

#### Fidelity to the daily caloric allowance

In the absence of the structured weekly dietary recall interview, the food intake data was less robust, as participants did not volunteer the depth of information that was elicited at the one-on-one interviews. On review of the group conversations, one participant reported some financial difficulty in sourcing the meals, as the Glucerna had been provided free of charge by the study and the responsibility to purchase meals, whether solid or liquid, was an unexpected burden. As a result, ultra-processed, hyper-dense foods were intermittently consumed. The same participant also had some difficulty determining non-exact portion sizes, e.g. medium orange. Additional information was gained when participants posted pictures of their meals—some of which were original recipes that they had created based on the solid meal plan (adaptation #3). A review by the study team determined that they were of the 840 kcal allowance.

### Change in clinical outcomes

Over the 12-week intervention period, one participant gained 0.4 kg whilst the remaining four participants lost 11.3–14.3 kg. Change in systolic BP ranged from − 21 to + 8 mmHg and diastolic BP from − 20 to + 7 mmHg. By the end of the intervention, all three participants who were hypertensive either reduced or discontinued their anti-hypertensive medications, and all five participants achieved diabetes remission based on both a FBG of < 7mmol/l and an A1C of < 6.5%. This compares to the previous face-to-face cohort where the average weight loss 6.8 kg and systolic BP and diastolic BP decreased by 10 mmHg and 8 mmHg, respectively. Sixty percent achieved T2DM remission by HbA1C threshold and 90% by FBG threshold.

## Discussion

This paper used the IDEA and the FRAME-IS to document the decision to adapt and to characterize the adaptations, respectively. We encountered two distinct challenges when utilizing the IDEA. The first challenge was the difficulty in our understanding the terms “core functions” and “core elements”.

Perez-Jolles et al. define a core function as the purpose of the health intervention [14]. They also added that “forms” were the specific strategies needed to fulfil the core functions. Miller et al., in the IDEA framework article, stated that “core elements should be conceptualized in terms of core functions that can take on varying forms” [10]. They also describe core elements as the “content, delivery mechanisms, or methods” of an intervention. Our understanding of this is that the elements are equal to the varying forms that the functions can take. A distinction on if core elements are functions or forms is important to the determination of IDEA component B—are core functions or elements known, and component C—can these core elements or functions be preserved. Given the inherent complexity in interpreting core elements and functions, we decided to conceptualize our current study using a combination of the Perez-Jolles framework and Miller definitions (Table 2). The core function was to stimulate weight loss. We expanded the framework at this point to include (i) forms that were non-adaptable—which we termed core elements, i.e. 12-week duration, 840 kcal allowance, and weekly monitoring—and (ii) forms that were adaptable—which we termed peripheral elements, i.e. who does the monitoring, where it is done, and the foods allowed in the calorie count.

**Table 2:** Core functions and forms in the BDRS2

Motivation/problem	Core function	Forms	
1. Reduce cardiovascular disease/high rates of obesity and T2DM	A. Stimulate weight loss	1. LCD	
		Core elements: • 12-week duration • 840 kcal • Weekly monitoring	Peripheral element: • Who monitors • The location for monitoring • The foods allowed

The second challenge was the lack of guidance on the de-implementation decision-making process in the absence of a voltage drop. In our study, the need to adapt was necessitated by a very specific trigger—a national lockdown due to COVID-19. The intervention results following the adaptation suggested a non-inferior outcome as compared to the original protocol. At the end of lockdown, how is a decision made on whether to continue the adapted protocol or to de-implement even in the face of non-inferiority?

Regarding the implementation strategy, the success of the shift in patient monitoring for the Barbados Diabetes Remission Study-2 (BDRS2) offers an additional option of patient contact and support for the larger healthcare system and is in keeping with the call from WHO for countries to “find innovative ways to ensure that essential services for non-communicable diseases continue, even as they fight COVID-19” [3]. The adaptations presented here maintained fidelity for the most part, to the key components of the original BDRS2 protocol whilst improving logistical fit to the current pandemic climate.

Although there were small numbers in our second cohort, there is early evidence that conversion to online monitoring has not harmed and has possibly improved our follow-up response rates. There was improved fidelity to the attendance schedule which was probably due to the simplicity and flexibility of home monitoring and data submission as compared to travelling to a community location for a specified time. On the other hand, we realize that the fidelity to the meal plan was stymied by the unexpected financial responsibility that was transferred to the participant, and the relatively truncated time that was allotted to instructing them on solid meal preparations. We also realized that in the absence of a structured interview, dietary recall data could be inadequate. Future studies should be mindful of these pitfalls and, wherever possible, dedicate additional time and resources to these areas.

The adapted protocol also potentially provides a solution to other limitations of the face-to-face meetings. Firstly, the number of participants accommodated on a weekly basis was limited by the space available. A fully online protocol or a hybrid system of face-to-face interspersed with mobile self-assessments would allow for the enrollment of larger numbers. This system also transfers a greater responsibility to participants during the low-calorie phase, which could potentially promote independence in the post-intervention era.

Although qualitative data from previous studies suggest that participants are amenable to the rigidity of the low-calorie liquid meal plan, the switch to comparably low-cost, locally sourced, solid recipes offer a solution to two limitations of the liquid formulation. Firstly, the commercially available liquid formulation is relatively costly, which could negatively impact scale-up and sustainability outside of a research setting where the drinks are not provided to clients, and secondly, switching to solid meals offers greater variety which could positively impact acceptability.

When switching meal plans, in addition to the caloric similarity, consideration must be given to the macronutrient content. Although comparable in carbohydrate and protein percentages, solid meals contained less fibre and sugar when compared to the liquid formulation; this could possibly impact the clinical outcome.

That participants were still able to lose weight during the adapted period without face-to-face formal support from a primary care physician, but with virtual informal support from peers participating in the intervention indicates that informal social support networks and self-efficacy should be considered in the design of an intervention. In this instance, the group dynamic that continued via WhatsApp during the COVID-19 lockdown protocols may have played a part in the success of self-monitoring as there was an element of accountability to others. This informal support can work in lieu of individualized attention from formal support networks, namely the primary care physician and other health care providers, thus reducing the burden on the healthcare system to provide formal support, when informal social support from peers allows for a similar level of accountability.

Research in other fields supports the findings from this study. Studies investigating virtual social support or comparing virtual treatments to face-to-face delivery have found the virtual option to be non-inferior or as effective as face-to-face [15, 16]. More specifically, work in diabetes prevention has found virtual interventions to be as effective as the in-person equivalent with similar outcomes reported in weight loss programmes [17, 18].

## Conclusion

These results suggest that the BDRS2 is adaptable from a fully face-to-face protocol to a fully online format whilst maintaining fidelity to the core intervention elements and producing similar clinical outcomes, thereby impacting health not just within the community but within the home.

Given that this was a small study, there is scope for comparative studies examining fully face-to-face vs fully virtual implementation strategies, and feasibility studies exploring the practicality of a hybrid implementation strategy. Additionally, proactive contextual modifications can be made to the existing protocol to increase the fit of the intervention to other community sites, e.g. service clubs. Utilizing the IDEA and the FRAME-IS in the planning and reporting of these adaptations will add scientific rigour to the growing body of adaptation literature, thereby facilitating comparisons among studies.

## Data Availability

The datasets used and/or analysed during the current study are available from the corresponding author on reasonable request.
